# Supplementary material for: Rapid turnover of life-cycle-related genes in the brown algae
Source: Genome Biol. 2019 Feb 14;20:35. doi: 10.1186/s13059-019-1630-6 (PMC6374913; doi:10.1186/s13059-019-1630-6)
Supplement: Supplementary file 3 — Protein alignments, corresponding Gblocks html file and CDS sequences for the lineage conserved gametophyte-biased genes. (ZIP 2109 kb) [file 13059_2019_1630_MOESM3_ESM.zip › Con_GA_alignments/Ec-20_002270.aa_ali.fasta-gb.htm]

Ec-20\_002270.aa.fasta.trans.aa\_ali.fasta


## Gblocks 0.91b Results

Processed file: **Ec-20\_002270.aa.fasta.trans.aa\_ali.fasta**  
Number of sequences: **2**  
Alignment assumed to be: **Protein**  
New number of positions: **577** (selected positions are underlined in blue)

```
                         10        20        30        40        50        60
                 =========+=========+=========+=========+=========+=========+
Ec-20_002270     MMALSVRTESFRGRGENTRNQGAGDAEQSRPSSSGGRLEQHLGAVESVDMPPAAAALVPS
MSTRG.36973.1    ------------------------------------------------------------
                                                                             


                         70        80        90       100       110       120
                 =========+=========+=========+=========+=========+=========+
Ec-20_002270     VPVASRTLGFPTGPRPTAVAVTPARINADARGERDVRGGGRAGGRDNGAAPTEHTRAFKG
MSTRG.36973.1    ------------------------------------------------------------
                                                                             


                        130       140       150       160       170       180
                 =========+=========+=========+=========+=========+=========+
Ec-20_002270     GSSGGASFGSPGSRVVVDEQHHLPTKRTSDARQQVVGGRRARDRKGGGRRRSVAEVGQTV
MSTRG.36973.1    ------------------------------------------------------------
                                                                             


                        190       200       210       220       230       240
                 =========+=========+=========+=========+=========+=========+
Ec-20_002270     GAEGNSGSLARGGGVVRGNADGVKERPRRVSSQLSFGSSVTEGGGGGGGAAGGGHRRGGQ
MSTRG.36973.1    ------------------------------------------------------------
                                                                             


                        250       260       270       280       290       300
                 =========+=========+=========+=========+=========+=========+
Ec-20_002270     RGGEVGENARRARLGTQDTKFRMQAGQSNTLVAVRLRPLLKHDREHVEVAKVLDHRVVVL
MSTRG.36973.1    ------------------------------------------------------------
                                                                             


                        310       320       330       340       350       360
                 =========+=========+=========+=========+=========+=========+
Ec-20_002270     MDPAKVNQEVDPLRVNRTREKRYAFDYVFDATATQRAVYENTTKFLIQGVVDGYNATVFA
MSTRG.36973.1    ------------------------------------------------------------
                                                                             


                        370       380       390       400       410       420
                 =========+=========+=========+=========+=========+=========+
Ec-20_002270     YGCTGAGKTYTMIGNGQEGHQGIMVLTLQDLFKQQRRASEVQGRRYSVTVSFLEVYNENI
MSTRG.36973.1    ------------------------------------------------------------
                                                                             


                        430       440       450       460       470       480
                 =========+=========+=========+=========+=========+=========+
Ec-20_002270     RDLLNDTGEFLDLREDPIKGPTVSGLAEVEARTPEEIMDLLHQGNQSRTQQATRANAASS
MSTRG.36973.1    ------------------------------------------------------------
                                                                             


                        490       500       510       520       530       540
                 =========+=========+=========+=========+=========+=========+
Ec-20_002270     RSHAVLQVVVESRDKAPGTVATMHIGKLSLVDLAGSERAAATQNRGVRLVEGANINRSLL
MSTRG.36973.1    ------------------------------------------------------------
                                                                             


                        550       560       570       580       590       600
                 =========+=========+=========+=========+=========+=========+
Ec-20_002270     ALGNCINALGERGNKGQFVPYRDSKLTRLLKDSLGGNCRTVMIANISGASSSFEETLNTL
MSTRG.36973.1    ---------------------------------------------HSGASSSFEETLNTL
                                                               ##############


                        610       620       630       640       650       660
                 =========+=========+=========+=========+=========+=========+
Ec-20_002270     KYANRAKNIKTNATRNSLDVNHHISEYVNLIGSLRTEITRLKHQLVRG---GRSGGGVGT
MSTRG.36973.1    KYANRAKNIKTNATRNSLDVNHHISEYVNLIGSLRTEITRLKHQLVRGAERGGGGGGIGR
                 ################################################   #########


                        670       680       690       700       710       720
                 =========+=========+=========+=========+=========+=========+
Ec-20_002270     GAGGDGVGSSR--SEAMVFSPSLKASTNSLFAPADDEQQELRGSRGKEITGSSERASLLL
MSTRG.36973.1    VIGGEGAASTNRSSETMVFSPSLKTSTNSLFACPENDG-EYQQELGAFPRGKRGESTEAC
                 #########    ###################       ############         


                        730       740       750       760       770       780
                 =========+=========+=========+=========+=========+=========+
Ec-20_002270     SPSSSPSAKLTNDVGSVGGVDAGAGSMMEAVDGAAGSGTSSRNGIDLAMVAGVRDAPASS
MSTRG.36973.1    TSSSRAAADVIDGNSGVIVEHSSRNAIRGGGGGDGAIAETSRGAGSLSSKSDLGPAPGGL
                   ###############               ############################


                        790       800       810       820       830       840
                 =========+=========+=========+=========+=========+=========+
Ec-20_002270     PPVS--MADGEGAVVSSPQDRDFVEEVREKIVENFRERMQLRRSLIELEDQNVQNSIEVG
MSTRG.36973.1    EPLSPPLATTEVAPPSSQEDKDFVEEVREKIVENFRERMQLRRSLIELEDQNVQNSIEVG
                 ####   #####################################################


                        850       860       870       880       890       900
                 =========+=========+=========+=========+=========+=========+
Ec-20_002270     KRQLSLVEWHGGRRLPQEAASDALRGLTRHGGWAEAVAEAAEAAAAGGRGDGSIISPPPP
MSTRG.36973.1    KRQLSLVEWHGGRRLPQEAASNALQGLTRHGGWAEAVAEAAEAAAGG-SASGESITPPPP
                 ###############################################    #########


                        910       920       930       940       950       960
                 =========+=========+=========+=========+=========+=========+
Ec-20_002270     EVRVAWRECEQLCSAISKNNETKRKISRRLRNNGRQAERFKADLDGKITGEDRNQLMRLQ
MSTRG.36973.1    EVKVAWRECEQLCSAISKNNETKRKISRRLRNNSRQAEKFKADLDGKITGEDRNQLMKLQ
                 ############################################################


                        970       980       990      1000      1010      1020
                 =========+=========+=========+=========+=========+=========+
Ec-20_002270     YRIGKLELDNMELEQSRIVHESAMRGKDLTIEKLQLQLAVRDRTIALQQSVLKEHGLDNV
MSTRG.36973.1    YRIGKLELDNMELEQSRIVHESAMRGKDLTIQKLQLQLAVRDRTIALQRSVLADHGLDGT
                 ############################################################


                       1030      1040      1050      1060      1070      1080
                 =========+=========+=========+=========+=========+=========+
Ec-20_002270     GFPGSVGVNQVGITDALDAFLPEGVVVGRTDSVGSTSFDDGDHGGDVHNGDDQDDGDDGD
MSTRG.36973.1    GSTDAVGVDQVGMTDALDAFLPERVVLGHVDSVGGEPID-GNIIITHDMNDASGEGDDGD
                 #######################################           ##########


                       1090      1100      1110      1120      1130      1140
                 =========+=========+=========+=========+=========+=========+
Ec-20_002270     SGVLIPRCRNGRRGGWGSSSSDSEEDSRQNTAAKMNGGGAKKDGPAGIAVIESGNINPSS
MSTRG.36973.1    QGAPIPRVG-RRIGGWGSSSDSEGGDPR--HAAQVAGDPENSD-SAGNAVRASG--AGSS
                 #######    #################   ############               ##


                       1150      1160      1170      1180      1190      1200
                 =========+=========+=========+=========+=========+=========+
Ec-20_002270     TQGGSASVSATDSAAGAEEDDKEKQEKSRHQKQQQQHQPRDRLEQMMETTLGRSTGQQWP
MSTRG.36973.1    ALARTHQVSSSLVSLASTSPAQVAAAPVTAERLQ--QQPRDSLEQMADTDVCGATG-QWP
                 #########                            ################### ###


                       1210      1220      1230      1240      1250      1260
                 =========+=========+=========+=========+=========+=========+
Ec-20_002270     KRRPSKTMSRTTRQTTHPPPPQPPLSSSSSLQPKPKYPQTRSRRRRVPSNTGGGGGGHLQ
MSTRG.36973.1    NRRPSKTTSRARQTMTFQASPQP--SSSLLQQKEPKQKQTRSRRRRAPSDASCGG-VHSQ
                 #######################  ##############################  ###


                       1270      1280      1290      1300      1310      1320
                 =========+=========+=========+=========+=========+=========+
Ec-20_002270     GDHDDDSIEDGLVVSSIIPDAASCVAPFHGPLPVSLAFGCSSDGLLAVPAGDDAFAAKGS
MSTRG.36973.1    EDHDDDSIEDGLIISSA--------------FP---------------------------
                 ################                                            


                       1330      1340      1350      1360      1370      1380
                 =========+=========+=========+=========+=========+=========+
Ec-20_002270     RARDERNLSLNDLAAVRGISVKQRQPVPKDRSPNPQNQPPSDSVAPSSAGFAGNSASGVP
MSTRG.36973.1    ---------------V---------------------------------RFAG-------
                                                                             


                       1390      1400      1410      1420      1430      1440
                 =========+=========+=========+=========+=========+=========+
Ec-20_002270     VTTWDGASGGDAAGGSGLPNGEGRTGNDLVIPNGPLAVSSSTGVGRMFQNQAQPPHPLKH
MSTRG.36973.1    --------------------GGGGTG------------TVSSG-----------------
                                                                             


                       1450      1460      1470      1480      1490      1500
                 =========+=========+=========+=========+=========+=========+
Ec-20_002270     GSSAYAEASARPAGLVADGPDSMGISGTSVALPWSRRQAPTSSCNAGGAIGAGADRRRLR
MSTRG.36973.1    ------------------------------------------------------------
                                                                             


                       1510      1520      1530      1540      1550      1560
                 =========+=========+=========+=========+=========+=========+
Ec-20_002270     REQTRAAAAGGVARSNWGSDAANTNVAAQGWGERSHGGDDVELRGRMGEDRPPMAHGVGT
MSTRG.36973.1    ------------------------------------------------------------
                                                                             


                       1570      1580      1590      1600      1610      1620
                 =========+=========+=========+=========+=========+=========+
Ec-20_002270     DAAEMIVVASALGAGRADEGEQREARFGSLANLKARMGVRCQQQQQQQQQPAEAAAGSGG
MSTRG.36973.1    ------------------------------------------------------------
                                                                             


                       1630      1640
                 =========+=========+========
Ec-20_002270     GGVASGERNTQESAGKDRGPRRTPHQAF
MSTRG.36973.1    ------VGGAKVVAG-------------
```

```
Parameters used
Minimum Number Of Sequences For A Conserved Position: 2
Minimum Number Of Sequences For A Flanking Position: 2
Maximum Number Of Contiguous Nonconserved Positions: 8
Minimum Length Of A Block: 10
Allowed Gap Positions: None
Use Similarity Matrices: Yes
```

```
Flank positions of the 16 selected block(s)
Flanks: [587  648]  [652  669]  [674  692]  [700  711]  [723  737]  [753  784]  [788  887]  [892  1059]  [1071  1087]  [1092  1108]  [1112  1123]  [1139  1149]  [1178  1196]  [1198  1223]  [1226  1255]  [1258  1276]  

New number of positions in Ec-20_002270.aa.fasta.trans.aa_ali.fasta-gb:  577  (35% of the original 1648 positions)
```
